# Supplementary material for: IGFBP7 Drives Resistance to Epidermal Growth Factor Receptor Tyrosine Kinase Inhibition in Lung Cancer
Source: Cancers (Basel). 2019 Jan 2;11(1):36. doi: 10.3390/cancers11010036 (PMC6356910; doi:10.3390/cancers11010036)
Supplement: Supplementary file 1 [file cancers-11-00036-s001.pdf]

Supplementary Methods: IGFBP7 Drives Resistance to Epidermal Growth Factor Receptor Tyrosine Kinase Inhibition in Lung Cancer

Shang-Gin Wu, Tzu-Hua Chang, Meng-Feng Tsai, Yi-Nan Liu, Chia-Lang Hsu, Yih-Leong Chang, Chong-Jen Yu and Jin-Yuan Shih

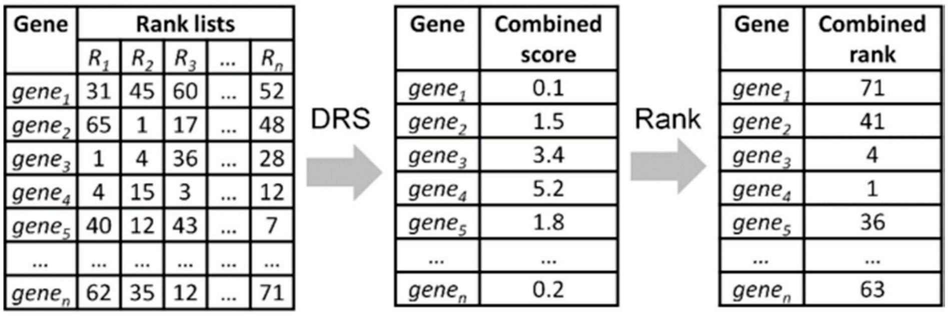

Figure S1. Illustration of the integration of multiple rank lists into a single score to identify TKI resistance-related genes.

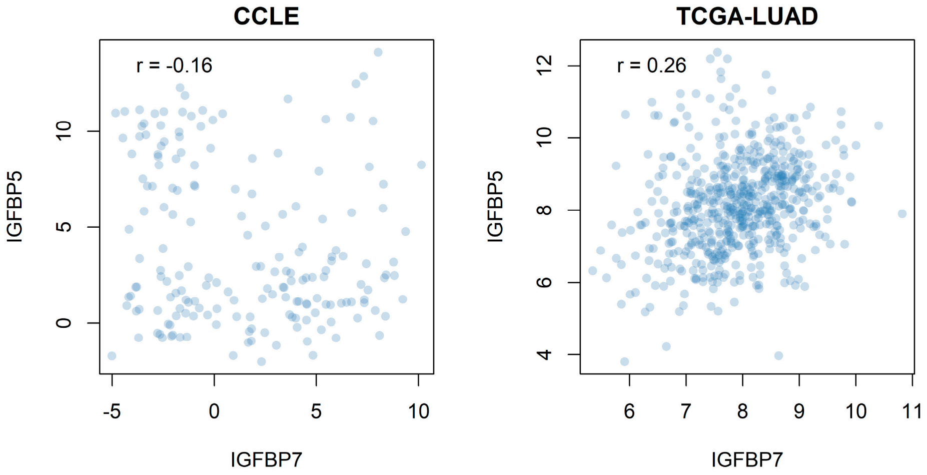

Figure S2. Gene expression correlation between *IGFBP7* and *IGFBP5* in lung cancer cell lines from Cancer Cell Line Encyclopedia (CCLE) and lung adenocarcinoma tissue from TCGA-LUAD datasets.

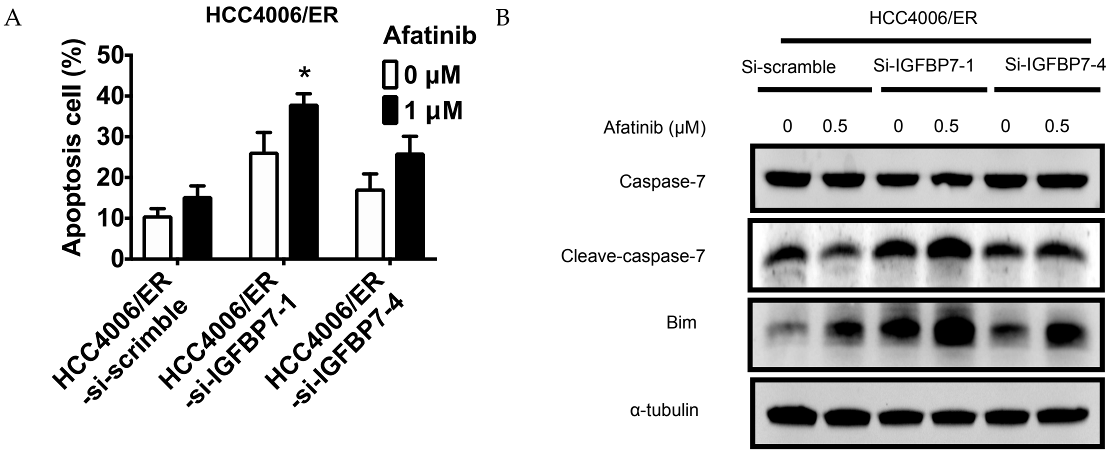

Figure S3. Knock-down IGFBP7 recovers EGFR-TKI sensitivity in EGFR-TKI-resistant cells by increasing apoptosis. (**A**) EGFR-TKI-resistant cells (HCC4006/ER) were transfected with different IGFBP7 small interfering RNAs (siRNAs) (si-IGFBP7-1, si-IGFBP7-4) or scramble siRNA (si-scramble).

The percentage of apoptotic cells was quantified after treatment with 1.0 μM afatinib for 24 h. The columns are the mean of three independent experiments. Error bars show the standard deviations for $n = 3$ independent experiments (* $p < 0.05$). (**B**) HCC4006/ER was exposed to 1.0 μM of afatinib for 24 h. Next, apoptosis markers, including cleaved-caspase-7 and BIM, were assayed by western blotting.

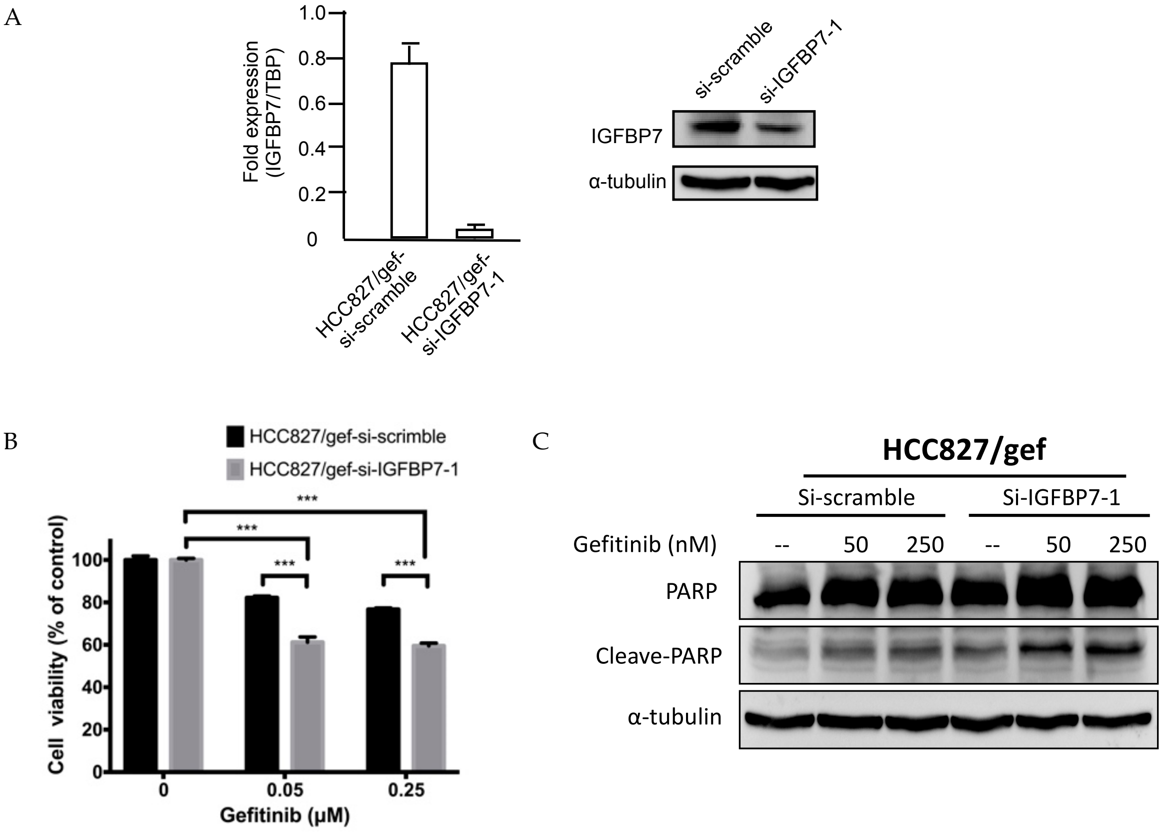

Figure S4. Knockdown of *IGFBP7* expression reversed EGFR-TKI resistance in HCC827/gef cells by enhancing EGFR-TKI-induced cleave-PARP expression (**A**) HCC827/gef cells were transfected with *IGFBP7* small interfering RNAs (siRNA; si-IGFBP7-1) or scramble siRNA (si-scramble). The effect of siRNAs was evaluated by quantitative RT-PCR (left) and western blot analysis (right). (**B**) Cellular viability of si-scramble and si-IGFBP7 transfectants was determined at different doses of gefitinib for 96 h using MTS assays. Error bars show the standard deviations for $n = 3$ independent experiments. (*** $p < 0.001$). (**C**) HCC827/gef was exposed to 50 and 250 nM of gefitinib for 24 h. Next, the apoptosis marker cleaved-PARP was assayed by western blot analysis.

Table S1 Gene expression profiles of TKI-sensitive and TKI-resistant human lung cancer cell lines from GEO.

No.	Source	TKI-Resistant Cells	TKI-Resistance Cell Lines	EGFR TKI	TKI-Sensitive Cells	TKI-Sensitive Cell Lines
**1**	GSE80344	GSM2124851	HCC4006-RC2.2-rep1	Erlotinib	GSM2124848	HCC4006
		GSM2124859	HCC4006-RC2.2-rep2		GSM2124856	HCC4006
**2**	GSE80344	GSM2124847	HCC827-RA1-rep1	Erlotinib	GSM2124850	HCC827
		GSM2124855	HCC827-RA1-rep2		GSM2124858	HCC827
**3**	GSE80344	GSM2124852	HCC827-RA2-rep1	Erlotinib	GSM2124850	HCC827
		GSM2124860	HCC827-RA2-rep2		GSM2124858	HCC827
**4**	GSE80344	GSM2124849	HCC827-RB1-rep1	Erlotinib	GSM2124850	HCC827
		GSM2124857	HCC827-RB1-rep2		GSM2124858	HCC827
**5**	GSE80344	GSM2124846	HCC827-RB1.1-rep1	Erlotinib	GSM2124850	HCC827
		GSM2124854	HCC827-RB1.1-rep2		GSM2124858	HCC827
**6**	GSE80344	GSM2124853	HCC827-RB2-rep1	Erlotinib	GSM2124850	HCC827
		GSM2124861	HCC827-RB2-rep2		GSM2124858	HCC827
**7**	GSE103350	GSM2768999	PC9GTR	Gefitinib	GSM2768998	PC9
**8**	GSE103350	GSM2769000	PC9OTR	Osimiertinib	GSM2768998	PC9
**9**	GSE103350	GSM2769002	HCC827GTR	Gefitinib	GSM2769001	HCC827
**10**	GSE103350	GSM2769003	HCC827OTR	Osimiertinib	GSM2769001	HCC827
**11**	GSE106765	GSM2850069	PC-9-AR	Afatinib	GSM2850068	PC9
**12**	GSE106765	GSM2850070	PC-9-OR	Osimiertinib	GSM2850068	PC9
**13**	GSE106765	GSM2850072	HCC827-AR	Afatinib	GSM2850071	HCC827
**14**	GSE106765	GSM2850073	HCC827-OR	Osimiertinib	GSM2850071	HCC827
**15**	GSE95558	GSM2516279	HCC827 ZDR3	Gefitinib	GSM2516278	HCC827

Table S2 IC_{50} values of EGFR-TKIs examined in this study are summarized. The growth inhibitory effects of EGFR-TKIs were measured using an MTS assay. The cells were treated with increasing doses of gefitinib, erlotinib, and afatinib for 96 h. IC_{50} values were calculated using SigmaPlot and are shown in the table.

Lung cancer cell lines	IC_{50} (μM)		
	Gefitinib (Iressa)	Erlotinib (Tarceva)	Afatinib (Giotrief)
**PC9**	0.04	0.02	<0.001
**PC9/gef**	6.01	4.66	0.68
**HCC827**	<0.0078	<0.0078	<0.0078
**HCC827/gef**	>10	>10	2.6714
**HCC4006**	0.01	0.06	<0.002
**HCC4006/ER**	20.05	28.97	2.98

Table S3 Clinical characteristics of the 102 patients with advanced lung adenocarcinoma administered EGFR-TKIs as first-line treatment.

Variable		Number of Patients	IGFBP7 IHC		*p*-Value
			Positive	Negative	
**Total No.**		102	53	49	
**Age median**		61.0	59.5	61.5	0.507 #
**range**		28.0–89.6	28.0–85.4	38.5–89.6	
**Sex**					
	**Female**	66	34	32	0.903
	**Male**	36	19	17	
**Smoking**					0.071
	**Non-/light smoker**	86	48	38	
	**Smoker**	16	5	11	
**EGFR-TKI**					0.346 *
	**gefitinib**	91	49	42	
	**erlotinib**	11	4	7	
**EGFR mutation**					0.628
	**Del-19**	47	22	25	
	**L858R**	48	27	21	
	**Other** β	7	4	3	

By Mann-Whitney U; * By Fisher exact test; β Other: 2 G719S, one G719A, one L861Q, one G719C+E790A, one G719S+G709A and one L858R+E709V; IHC: Immunohistochemical staining.

Table S4 Clinical characteristics of 75 patients with advanced lung adenocarcinoma administered EGFR-TKIs as first-line treatment (cut-off point was the median value of IGFBP7: 544.96 ng/mL).

Caption	Patient No.	Serum IGFBP7 level		*p*-Value
		High	Low	
**Total No.**	75	38	37	
**Mean years of age**	63.8	66.1	61.1	0.053 *
**range**	42.7–88.5	44.1–88.5	42.7–83.6	
**Sex**				0.110
**Female**	53	30	23	
**Male**	22	8	14	
**Smoking**				0.708
**Nonsmokers**	64	33	31	
**Former/current smokers**	11	5	6	
**T**				0.629
**1**	3	1	2	
**2**	9	2	7	
**3**	1	0	1	
**4**	16	7	9	
**N**				0.186
**0**	1	0	1	
**1**	4	3	1	
**2**	11	2	9	
**3**	13	5	8	
**EGFR-TKI**				0.430 §
**gefitinib**	68	33	35	
**erlotinib**	7	5	2	

§ by Fisher's Exact test; * by Mann-Whitney U test.

Table S5. List of primers for quantitative RT-PCR.

Primers	Sequences
**IGFBP7**	**F**: 5′-ACTGGCTGGGTGCTGGTA-3′
	**R**: 5′-TGG ATG CAT GGC ACT CAT A-3′
**SPANXA1**	**F**: 5′-AACGAGGCCAACGAGATGAT-3′
	**R**: 5′-CTAGTATGGTCGAGGACTCAGATGTT-3′
**HRASLS**	**F**: 5′-CTGTACTTGGGTGATGGTTACGTTA-3′
	**R**: 5′-AGACTTGGCGCTTGTAAAGGAC-3′
**RNF182**	**F**: 5′-TCTTAGAGGCAGGACTTGATGA-3′
	**R**: 5′-AAGGCCACATGAAGGGTTC-3′
**SPANXC**	**F**: 5′-CAACGAGGTGAATGAGACGA-3′
	**R**: 5′-TGGTCGAGGACTCAGATGTTT-3′
**LY6D**	**F**: 5′-CGAACACAGTGGAGCCTCTGA-3′
	**R**: 5′-AGCTTCTCATTGCACAGGTCC-3′

Table S6. List of antibodies for western blot analysis.

Primary antibody	Company	Dilution
**IGFBP7**	R&D Systems	1:2000
**PARP**	Cell Signaling	1:1000
**caspase-3**	Cell Signaling	1:1000
**caspase-7**	Cell Signaling	1:1000
**Bim**	Cell Signaling	1:1000
**pIGF1R**	Santa Cruz	1:500
**IGF1R**	GeneTex	1:1000
**pAkt**	Cell Signaling	1:1000
**Akt**	Cell Signaling	1:2000
**pErk**	Cell Signaling	1:1000
**Erk**	Cell Signaling	1:2000
**α-tubulin**	Millipore	1:3000
**β-actin**	Millipore	1:3000

Supplementary Method

Data sources and gene ranking

We collected gene the expression profiles of TKI–sensitive and TKI-resistant human lung cancer cell lines from the GEO with accession numbers GSE80344, GSE10335, GSE106765, and GSE95558 consisting of 15 comparisons of TKI-resistant to TKI-sensitive cell lines (Table S1). The raw data from GSE106765 were processed using R affy packages and normalized by Frozen Robust Multi-Array Analysis implemented in the frma packages [1]. The raw data from GSE80344 were processed, including background correction, quantile normalization, and summarization, using the limma package in R [2]. The read counts from GSE10335 and GSE95558 were normalized by the trimmed mean of M-values normalization method (TMM) implemented by edgeR [3]. For each comparison, NOISeq [4] was performed to obtain the logarithm fold-change (logFC) and probability of differential expression (*prob*) between TKI-resistant and TKI-sensitive cells. The TKI resistance-related score was defined as follows:

$$Score = sign(logFC) \times prob$$

Where sign () is the sign function. Genes were ranked in descending order based on this score.

Rank list fusion

The discounted rating system was used to combine these ranking lists into a single score [5]. Briefly, for each rank list, genes were categorized into ten equal-size bins based on their rank positions and assigned ratings ranging from 10 to 1. A higher rating indicates that the gene was more

relevant to TKI resistance. In the next step, the discounted rating for each gene (dr_i) was calculated using the following formula:

$$dr_i = \frac{ranting_i}{log_2(r_i + 1)}$$

Where $rating_i$ and r_i are the rating and rank position of the investigated gene in experiment i, respectively. Finally, the combined score for the investigated gene was the mean value of the eight highest discounted ratings of the experiments, and the genes were ranked according to this score.

References

1. McCall, M.N.; Jaffee, H.A.; Irizarry, R.A. fRMA ST: Frozen robust multiarray analysis for Affymetrix Exon and Gene ST arrays. *Bioinformatics* **2012**, *28*, 3153–3154.
2. Ritchie, M.E.; Phipson, B.; Wu, D.; Hu, Y.; Law, C.W.; Shi, W.; Smyth, G.K. Limma powers differential expression analyses for RNA-sequencing and microarray studies. *Nucleic Acids Res.* **2015**, *43*, e47.
3. Robinson, M.D.; McCarthy, D.J.; Smyth, G.K. edgeR: A Bioconductor package for differential expression analysis of digital gene expression data. *Bioinformatics* **2010**, *26*, 139–140.
4. Tarazona, S.; Furio-Tari, P.; Turra, D.; Pietro, A.D.; Nueda, M.J.; Ferrer, A.; Conesa, A. Data quality aware analysis of differential expression in RNA-seq with NOISeq R/Bioc package. *Nucleic Acids Res.* **2015**, *43*, e140.
5. Li, Y.; Patra, J.C. Integration of multiple data sources to prioritize candidate genes using discounted rating system. *BMC Bioinformatics* **2010,** *1*, doi: 10.1186/1471-2105-11-S1-S20.

© 2019 by the authors. Licensee MDPI, Basel, Switzerland. This article is an open access article distributed under the terms and conditions of the Creative Commons Attribution (CC BY) license (http://creativecommons.org/licenses/by/4.0/).
